# Molecular Detection of *Diphyllobothrium nihonkaiense* in Humans, China

**DOI:** 10.3201/eid2002.121889

**Published:** 2014-02

**Authors:** Shanhong Chen, Lin Ai, Yongnian Zhang, Jiaxu Chen, Weizhe Zhang, Yihong Li, Maki Muto, Yasuyuki Morishima, Hiromu Sugiyama, Xuenian Xu, Xiaonong Zhou, Hiroshi Yamasaki

**Affiliations:** Chinese Center for Disease Control and Prevention, Shanghai, China (S. Chen, L. Ai, Y. Zhang, J. Chen, X. Xu, X. Zhou);; Harbin Medical University, Harbin, Heilongjiang Province, China (W. Zhang, Y. Li);; National Institute of Infectious Diseases, Tokyo, Japan (M. Muto, Y. Morishima, H. Sugiyama, H. Yamasaki)

**Keywords:** cestodiasis, diphyllobothrium, diphyllobothrium infection, nihonkaiense, latum, diphyllobothriosis, tapeworm, proglottid, plerocoercoid, strobila, parasite, China

## Abstract

The cause of diphyllobothriosis in 5 persons in Harbin and Shanghai, China, during 2008–2011, initially attributed to the tapeworm *Diphyllobothrium latum*, was confirmed as *D. nihonkaiense* by using molecular analysis of expelled proglottids. The use of morphologic characteristics alone to identify this organism was inadequate and led to misidentification of the species.

Diphyllobothriosis is a fishborne cestodiasis caused by infection with adult tapeworms belonging to the genus *Diphyllobothrium* Cobbold, 1858 (*1*–*5*); the most frequent etiologic agents are *D. latum* and *D. nihonkaiense*. Humans are infected by ingesting raw or undercooked fish infected with larval plerocercoids. Adult tapeworms can grow to ≈2–10 m in length in the human small intestine (*1**–**6**).* Despite the large size of the tapeworms, clinical symptoms can be absent or mild and include mild abdominal pain, watery diarrhea, and abdominal discomfort (*3*–*7*). *D. latum* infection can also cause vitamin B_12_-deficiency anemia (*5*).

Diphyllobothriosis caused by *D. nihonkaiense* has been extensively reported in Japan (*3**,**4*), but it has also occurred autochthonously in South Korea (*8*) and the Far Eastern Federal District of Russia (originally reported as *D. klebanovskii* infection [*9**]*). Sporadic cases have been reported in Europe (*6*), North America (*10*), and New Zealand (*7*) in recent years.

In mainland China, 15 cases of diphyllobothriosis among humans have been reported since the first report in 1927 through 2012; the etiologic species was identified as *D. latum* by morphologic characteristics (*11**–**13*; Table) and molecular markers (*14**,**15*). No cases of diphyllobothriosis had been reported in large cities such as Beijing and Shanghai during 1954–2007 (*11*). However, we confirm 4 cases of D. nihonkaiense infection in humans in Shanghai, previously identified as *D. latum* infection, during 2008–2011, as well as 1 case in the moderately populous city of Harbin in Heilongjiang Province.

## The Study

We examined 5 recent infections of humans with *Diphyllobothrium* spp. (Table, cases 12, 16–19) that occurred in China. Each case had been originally reported as a D. latum infection on the basis of morphologic identification only. Case 12 was reported in Harbin City, Heilongjiang Province, in 2009 (*13*). The 4 cases reported in Shanghai were diagnosed at the National Institute for Parasitic Diseases, Shanghai, on the basis of morphologic features of passed strobila. Case-patient 16 lived in Japan, but it was suggested that he acquired the tapeworm in Shanghai where he had frequently eaten raw salmon. Case-patient 17 was a 10-year-old girl from Japan. Whether she became infected in Shanghai or Japan was unclear because of lack of information. Case-patients 18 and 19 acquired the infection in Shanghai because they had never been abroad. 

**Table:** Cases of infection with Diphyllobothrium species in humans reported in mainland China, 1927–2012*

Case no.	Residence/place of eating fish, if different	Proglottids expelled	Suspected source of infection	Proglottid specimen fixative	Morphologic identification	Molecular identification	GenBank accession no. for *cox1* gene	Reference
1–4	Heilongjiang	NA	NA	NA	D. latum	NT	NA	(*11*)
5	Beijing	NA	NA	NA	D. latum	NT	NA	(*11*)
6	Shanghai	NA	NA	NA	D. latum	NT	NA	(*11*)
7	Beijing (returned from United States)	NA	NA	NA	D. latum	NT	NA	(*11*)
8	Guangzhou (returned from Argentina)	NA	NA	NA	D. latum	NT	NA	(*11*)
9	Heilongjiang	NA	Raw fish	NA	D. latum	NT	NA	(*11*)
10	Jilin	NA	Raw fish	NA	D. latum	NT	NA	(*11*)
11	Fujian (lived in Yokohama, Japan, until 1996)	2003 Jan	Plecoglossus altivelis	NA	D. latum	NT	NA	(*12*)
12	Heilongjiang	2009 Jan	Raw fish	10% formalin	D. latum	D. nihonkaiense	AB684625	(*13*) and this study
13	Heilongjiang	NA	Salmon	NA	D. latum	D. latum	NA	(*14*)
14	Jilin	NA	Salmon	NA	D. latum	D. latum	NA	(*14*)
15	Shanghai, 2008-2011/Japan, China	2011 Dec	Raw sea and freshwater fish	NA	D. latum	D. latum	NA	(*15*)
16	Shanghai/Japan (returned from Japan in June 2008; ate raw salmon in Shanghai)	2008 Oct	Raw salmon	10% formalin	D. latum	D. nihonkaiense	AB684624	This study
17	Shanghai	2011 Sep	Raw salmon	70% ethanol	D. latum	D. nihonkaiense	AB684621	This study
18	Shanghai. Ate raw salmon in April 2011	2011 Jun	Raw salmon	70% ethanol	D. latum	D. nihonkaiense	AB684622	This study
19	Shanghai. Ate raw salmon in 2011	2011 Jul	Raw salmon	70% ethanol	D. latum	D. nihonkaiense	AB684623	This study

*Identification of Diphyllobothrium spp. was performed by morphologic identification alone in cases 1–11; organisms in each case were identified as D. latum. Cases 12–19 were assessed by morphologic and molecular identification; morphologic identification of all specimens was D. latum. Molecular identification varied from morphologic findings in 5 of 8 tested specimens. NA, not available; NT, not tested.

**Figure 1:**
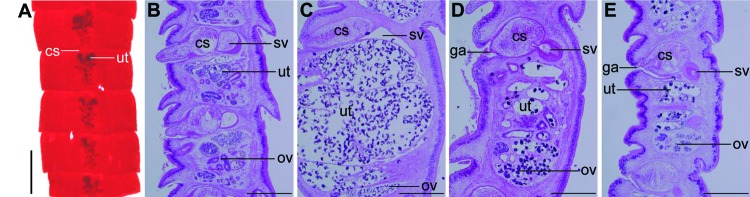
Diphyllobothriid samples examined in the present study, China, 2008–2012. A) Proglottids stained with acetic acid–carmine from case-patient 12. B–E) Sagittal sections of proglottids stained with hematoxylin-eosin from case-patients 16–19. cs, cirrus sac; ut, uterus; sv, seminal vesicle; ov, ovary; ga, genital atrium. Scale bar in panel A represents 2 mm; scale bars in panels B–E represent 500 μm.

Because all patients in Shanghai had eaten raw salmon, we decided to re-examine how the causative *Diphyllobothrium* spp.were identified. *D. latum* infection is associated with consumption of freshwater fish such as perch (*Perca* spp.), not Pacific salmon (*Oncorhynchus keta*, *O. masou*) and Atlantic salmon (*Salmo salar*) in the Northern Hemisphere (*1**–**5*). To expand diagnostic parameters and clarify the point of misidentification, we re-identified *Diphyllobothrium* spp. by examining the tapeworms’ morphologic features and using a molecular marker. In a sample from case-patient 12, only proglottids stained with acetic acid–carmine were available for testing by both methods (Figure 1, panel A). Proglottids obtained from 4 case-patients in Shanghai were preserved in either 10% formalin (case-patient 16) or 70% ethanol (case-patients 17–19) after collection (Table). Parts of the proglottids were embedded in paraffin, and sagittal sections were prepared for morphologic observation.

For molecular identification of the *Diphyllobothrium* spp., genomic DNA samples were extracted from specimens by using a DNeasy Blood & Tissue Kit (QIAGEN, Hilden, Germany). In specimens from case-patients 17–19, the mitochondrial cytochrome *c* oxidase subunit 1 gene (*cox1*, 1,566 bp) was amplified by PCR by using *Ex Taq* DNA polymerase (Takara Bio, Shiga, Japan) (*7*). In formalin-fixed samples of proglottids from case-patients 12 and 16, DNA degradation caused by the fixative meant that only shorter *cox1* fragments (249 bp, corresponding to sites 880–1128 of *cox1*) could be amplified successfully by PCR by using KOD FX DNA polymerase (Toyobo, Osaka, Japan). DNA sequencing of amplicons was performed with a 3100-*Advant* Genetic Analyzer or 3730 xl DNA Analyzer (Life Technologies, Foster City, CA, USA). Phylogenetic analysis was performed by the maximum likelihood method (MEGA 5.05, http://megasoftware.net/mega.php) and Bayesian inference (MrBayes version 3.1.2, http://mrbayes.sourceforge.net/). Clades were assessed by bootstrap resampling (1,000 replicates) and a posterior probability (10^6^ generations) for the maximum likelihood and Bayesian inference trees, respectively. *Diphyllobothrium* spp. isolated from case-patients 12 and 16 were identified on the basis of sequence identity (%) by performing a BLAST (http://blast.ncbi.nlm.nih.gov/Blast.cgi) analysis of a DNA Data Bank of Japan (http://www.ddbj.nig.ac.jp). 

Accurately identifying the *Diphyllobothrium* spp. isolated from case-patient 12 on the basis of morphologic features alone was difficult (Figure 1, panel A). In Figure 1, panels B–E show the sagittal sections of the proglottids from case-patients 16–19. The angle formed by the cirrus sac and the anterior–posterior axis of the proglottids was used as a criterion for differentiating *D. latum* from *D. nihonkaiense* (*1*), even though this criterion is not considered definitive: the angle is usually horizontal in D. latum, but oblique in D. nihonkaiense. Nonetheless, in this study, on the basis of morphologic criteria, tapeworms from case-patients 16, 17, and 19 were identified as *D.* latum (Figure 1, panels B, C, and E) and the tapeworm found in case-patient 18 was identified as *D.* nihonkaiense (Figure 1, panel D). 

**Figure 2:**
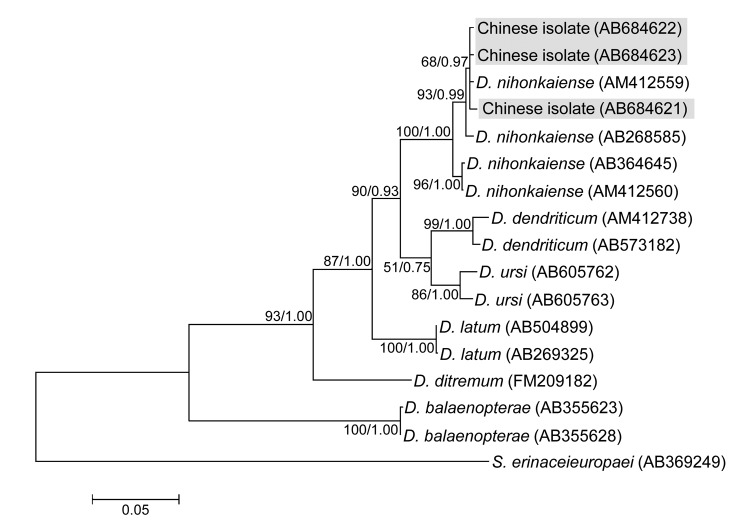
Phylogenetic tree constructed by using the maximum likelihood algorithm (Kimura’s 2-parameter model) on the basis of the complete *cox1* sequences of isolates from *Diphyllobothrium* species found in persons in China and related *Diphyllobothrium* species. Numbers at nodes are bootstrap values (1,000 replicates) and posterior probabilities (10^6^ generations) for maximum likelihood and Bayesian inference, respectively. *Spirometra erinaceieuropaei* was used as an outgroup. Scale bar indicates the number of base substitutions per site.

Phylogenetic trees based on the complete *cox1* nucleotide sequences showed the same topologies in maximum likelihood and Bayesian inference analyses, implying that the 3 isolates from persons in China (case-patients 17–19; GenBank accession numbers AB684621–AB648623) are *D. nihonkaiense* (Figure 2). The 2 isolates (AB684625 and AB684624) from case-patients 12 and 16, respectively, were excluded from the analysis because they produced smaller PCR products, but they were identified as *D. nihonkaiense* on the basis of their 99%–100% sequence identity to *D. nihonkaiense*.

The 5 *Diphyllobothrium* spp. tapeworms examined in this study were previously identified as *D. latum* on the basis of morphologic characteristics, as were 3 of the 5 when we re-examined their morphologic characteristics. However, the 5 etiologic agents were confirmed as *D. nihonkaiense* by molecular analysis. This discrepancy in the identity of these agents may be attributed to the morphologic similarities between the species and the century-long confusion between the parasite *D. latum* and the parasite that caused human diphyllobothriosis associated with the consumption of Pacific salmon in Japan (*1**–**3*). Diphyllobothriosis caused by *D. nihonkaiense* has also been reported in South Korea (*8*) and in the Far Eastern Federal District of Russia (*9*) and is considered to be autochthonous and linked to the consumption of wild Pacific salmon in these regions. Therefore, some cases of diphyllobothriosis reported in mainland China were probably caused by infections with *D. nihonkaiense*; case-patient 12 (*13*) in this study is considered to have had such a case. However, a recent report stating that the causative species of 2 diphyllobothriosis cases in northeastern China was *D. latum* which suggests that *D. latum* is also indigenous to mainland China (*14*). 

## Conclusions

We confirmed human diphyllobothriosis caused by *D. nihonkaiense* in mainland China by using a mitochondrial DNA marker. Reassessment of a case in Harbin revealed that some, if not all, of the autochthonous diphyllobothriosis cases were likely initially misdiagnosed as *D. latum* infection because of morphologic similarities between *D. nihonkaiense* and *D. latum* tapeworms. Consequently, molecular analysis is indispensable not only for avoiding diagnostic confusion among *Diphyllobothrium* spp., but also for facilitating the acquisition of reliable epidemiologic and epizootic information and improving clinical relevance and preventive controls for diphyllobothriosis. 

Information on diphyllobothriosis and warnings of the potential risks associated with infection by its local species should be disseminated to food handlers, restaurant owners, physicians, and consumers. Because we cannot determine with certainty whether previous diphyllobothriosis cases in mainland China were caused by *D. latum* or *D. nihonkaiense*, identification of *Diphyllobothrium* spp. should be performed with care. In addition, studies on the distribution and sources of infection of *D. latum* and *D. nihonkaiense* on mainland China should be undertaken.

## References

[1] Kamo H. Guide to identification of diphyllobothriid cestodes (ed., K. Hirai). 1999. Gendai Kikaku: Tokyo, Japan; 1999. [in Japanese].

[2] Dick TA, Nelson PA, Choudhury A. Diphyllobothriasis: update on human cases, foci, patterns and sources of human infections and future considerations. Southeast Asian J Trop Med Public Health. 2001;32(Suppl 2):59–76 . 12041607

[3] Yamane Y, Shiwaku K. *Diphyllobothrium nihonkaiense* and other marine-origin cestodes. In: Otsuru M, Kamegai, S, Hayashi S, editors. Progress of medical parasitology in Japan, vol. 8. Tokyo (Japan): Meguro Parasitological Museum;2003. p. 245–59.

[4] Arizono N, Yamada M, Nakamura-Uchiyama F, Ohnishi K. Diphyllobothriasis associated with eating raw Pacific salmon. Emerg Infect Dis. 2009;15:866–70. 10.3201/eid1506.090132 19523283 PMC2727320

[5] Scholz T, Garcia HH, Kuchta R, Wicht B. Update on the human broad tapeworm (genus *Diphyllobothrium*), including clinical relevance. Clin Microbiol Rev. 2009;22:146–60 . 10.1128/CMR.00033-08 19136438 PMC2620636

[6] Yéra H, Estran C, Delaunay P, Gari-Toussaint M, Dupoy-Camet J, Marty P. Putative *Diphyllobothrium nihonkaiense* acquired from a Pacific salmon (*Oncorhynchus keta*) eaten in France: genomic identification and case report. Parasitol Int. 2006;55:45–9. 10.1016/j.parint.2005.09.004 16243582

[7] Yamasaki H, Kuramochi T. A case of *Diphyllobothrium nihonkaiense* infection possibly linked to salmon consumption in New Zealand. Parasitol Res. 2009;105:583–6. 10.1007/s00436-009-1468-z 19444469

[8] Jeon HK, Kim KH, Huh S, Chai JY, Min DY, Rim HJ, Morphologic and genetic identification of *Diphyllobothrium nihonkaiense* in Korea. Korean J Parasitol. 2009;47:369–75. 10.3347/kjp.2009.47.4.369 19967084 PMC2788715

[9] Arizono N, Shedko M, Yamada M, Uchikawa R, Tegoshi T, Takeda K, Mitochondrial DNA divergence in populations of the tapeworm *Diphyllobothrium nihonkaiense* and its phylogenetic relationship with *Diphyllobothrium klebanovskii.* Parasitol Int. 2009;58:22–8. 10.1016/j.parint.2008.09.001 18835460

[10] Wicht B, Scholz T, Kuchta R. First record of human infection with the tapeworm *Diphyllobothrium nihonkaiense* in North America. Am J Trop Med Hyg. 2008;78:235–8 . 18256421

[11] Wu GL. Cestodes. In: Human parasitology, 3rd ed. Beijing: Peoples Medical Publishing House; 2005. p. 582–6. [in Chinese].

[12] Chen BJ, Li LS, Ling JX, Lin YS, Li YR. A case of human diphyllobothriasis after eating *Plecoglossus altivelis* [in Chinese]. Journal of Tropical Diseases and Parasitology. 2005;3:136.

[13] Li YH, Wen JS, Shu J, Zhang WZ. A human case caused by *Diphyllobothrium latum* in Heilongjiang Province [in Chinese]. Chinese Journal of Parasitology and Parasitic Diseases. 2009;27:296, 302. 20066981

[14] Guo AJ, Liu K, Gong W, Luo XN, Yan HB, Zhao SB, Molecular identification of *Diphyllobothrium latum* and a brief review of diphyllobothriosis in China. Acta Parasitol. 2012;57:293–6. 10.2478/s11686-012-0036-3 22875677

[15] Li H, Chen SH, Zhang YN, Ai L, Chen JX. A human case report of *Diphyllobothrium latum* at Shanghai, China. Journal of Animal and Veterinary Advances. 2012;11:3073–5. 10.3923/javaa.2012.3073.3075

